# Task-Dependent Cortical Oscillatory Dynamics in Functional Constipation

**DOI:** 10.3390/s26010211

**Published:** 2025-12-29

**Authors:** Jianhua Li, Hui Yang, Mingwei Xu, Yiman Wu, Xiaokai Shou, Zhihui Huang, Yan Hao, Fangchao Wu, Weishuyi Ruan, Ying Zhang, Zhengzhe Cui, Yina Wei

**Affiliations:** 1 Department of Rehabilitation Medicine, Sir Run Run Shaw Hospital, School of Medicine, Zhejiang University, Hangzhou 310016, China; jianhua_li@zju.edu.cn (J.L.); srrsh_haoyan@zju.edu.cn (Y.H.); 3414030@zju.edu.cn (F.W.); ruanweishuyi@zju.edu.cn (W.R.); zy0327@zcmu.edu.cn (Y.Z.); 2 Pelvic Floor Dysfunction Diagnosis, Treatment and Rehabilitation Center, Sir Run Run Shaw Hospital, School of Medicine, Zhejiang University, Hangzhou 310016, China; 3 Key Laboratory for Biomedical Engineering of Ministry of Education, Department of Biomedical Engineering, College of Biomedical Engineering and Instrument Science, Zhejiang University, Hangzhou 310027, China; hui.yang@zju.edu.cn (H.Y.); 12515047@zju.edu.cn (Y.W.); 4 Research Center for Frontier Fundamental Studies, Zhejiang Lab, Hangzhou 311100, China; 5 Department of Electrical & Computer Engineering, College of Engineering, University of Washington, Seattle, WA 98195, USA; xmw03@uw.edu; 6 Zhejiang Provincial Engineering Research Center of Rehabilitation Medicine Brain-Computer Interface Technology and Equipment, Hangzhou 311200, China; 211122020118@zjut.edu.cn (X.S.); 11325064@zju.edu.cn (Z.C.); 7 Department of Gastroenterology, Sir Run Run Shaw Hospital, School of Medicine, Zhejiang University, Hangzhou 310016, China; zhihui808@zju.edu.cn; 8 The College of Mechanical Engineering, Zhejiang University of Technology, Hangzhou 310023, China; 9 Institute for Brain and Intelligence, Fudan University, Shanghai 201203, China; 10 College of Biomedical Engineering, Fudan University, Shanghai 200433, China

**Keywords:** functional constipation, brain–gut axis, electroencephalography (EEG), cortical oscillations

## Abstract

Functional constipation (FC) is a common functional gastrointestinal disorder thought to arise from the brain–gut axis dysfunction, yet direct human neurophysiological evidence is lacking. We recorded high-density electroencephalography (EEG) data in 21 FC patients and 37 healthy controls across resting, cognitive, and defecation-related tasks. We observed that FC patients displayed a consistent, task-dependent signature compared with healthy controls. At the regional level, FC patients exhibited increased alpha during both resting and defecation-related tasks, reduced temporal gamma during defecation-related tasks, as well as elevated temporal theta during the cognitive task. At the global level, we found altered network properties, such as global efficiency in the delta and beta band networks during resting and defecation-related tasks. These findings establish a direct neurophysiological link between specific, condition-dependent perturbations in cortical rhythm activity and FC pathophysiology. Our work implicates the brain–gut axis in symptom generation and opens a path toward EEG-based biomarkers and targeted neuromodulatory therapies.

## 1. Introduction

Functional constipation (FC) is a common functional gastrointestinal disorder [1] characterized by core clinical manifestations such as defecatory difficulty, reduced defecation frequency, and sensation of incomplete evacuation. Its diagnosis is based on the Rome IV criteria and requires the exclusion of organic pathologies [2]. The global prevalence among adults is approximately 10–20%, and the pooled prevalence in pediatric populations reaches 14.4% (95% confidence interval: 11.2–17.6) [3]. Given its high incidence, FC must be recognized not merely as a routine clinical condition, but as a public health problem requiring collaborative efforts between clinicians and researchers.

The FC is multifactorial, involving the interaction of multiple physiological and pathological factors [4]. The brain–gut axis, as a key regulatory system that connects the central nervous system and the gastrointestinal tract, plays a pivotal role in the occurrence and progression of FC. The brain–gut axis modulates intestinal motility, visceral sensitivity, and ultimately stool excretion by regulating neural signaling transmission [5]. Therefore, the abnormal transmission of brain–gut axis signals is considered a critical driver of FC pathogenesis [6]. Notably, exploring the cerebral neural correlates in FC is of critical clinical importance. Within the brain–gut axis, brain signals serve as core functional carriers. Their decoding allows for the identification of specific neuromodulatory targets, which in turn facilitates targeted interventions to rectify aberrant cerebral regulatory signals in the brain–gut axis, thereby enhancing the efficacy of restoring physiological defecation.

Existing magnetic resonance imaging (MRI)-based investigations have demonstrated that FC patients exhibit significant structural and functional alterations in specific brain regions, including those involved in visceral sensorimotor (SM) function, default mode (DM) network, cognitive control (CC), and emotional regulation [7,8]. Among these altered brain regions, the anterior insula (aINS) shows more pronounced changes. As a core node for visceral sensory reception and processing as well as a key brain region of the salience network, the aINS is thought to be involved in integrating relevant signals. Altered structural and functional connectivity patterns of the aINS in FC patients may affect the integration of visceral sensory and emotional signals [9,10]. These changes are also associated with the severity of constipation symptoms and may weaken the brain’s ability to regulate intestinal motility [9]. In addition, the rostral anterior cingulate cortex (rACC), another core node of the salience network, shows reduced within-module nodal degree and nodal efficiency [10].

In addition, the altered whole-brain network organization is also a key pathophysiological feature of FC. Analysis of functional connectivity for FC patients reveals characteristics of brain functional networks [11], including altered emotion-autonomic integration networks, self-monitoring networks, and visual-sensorimotor networks. These network-level changes have been associated with defecation reflex dysregulation and emotional disorders [12,13]. Furthermore, previous studies have shown that elderly FC patients exhibit altered topological properties in limbic system networks, the supplementary motor area, and DM networks [13]. These findings suggest changes in viscerosensory-visceromotor integration and emotion regulation networks within the brain–gut axis framework. These alterations in brain networks, characterized by disrupted static coupling and topological features, are mechanistically associated with altered visual perception, emotional disorders, sensorimotor dysfunction, and attention deficits [12,14].

Existing neuroimaging research has extensively observed altered static resting-state functional connectivity in FC, positioning it as a brain–gut interaction disorder [7,8,9,10,12,13,14]. However, these studies are limited by their reliance on the resting state and fail to probe cognitive or motor processes relevant to FC pathology [15]. A further limitation is the predominant use of MRI, whose relatively low temporal resolution constrains the analysis of neural dynamics [16].

While Electroencephalogram (EEG) offers millisecond temporal resolution [17] ideal for probing brain–gut interactions, its application to FC remains largely untapped. Existing EEG studies have provided fragmented insights, reporting attentional deficits [18], implicit cognitive dysfunction [19], and peripheral sensory pathway alterations [20] in FC patients. However, these pioneering studies have provided initial insights but are constrained by a predominant reliance on event-related potentials (ERPs), and homogeneous task paradigms, thus failing to provide a comprehensive view of brain activity in FC.

To address these gaps, we collected EEG to capture real-time neural dynamics across a multi-task paradigm, including resting, cognitive, and defecation-related conditions from both FC patients and healthy controls. This design assesses brain activity under states that directly modeled on the daily, symptom-provoking experiences of FC patients. We analyzed EEG signals in both time-frequency and spatial domains to capture both localized neural dynamics and global network topology. By comparing patients and controls, our approach offers a more comprehensive characterization of brain activity in FC, thereby providing a complementary perspective on its underlying neural mechanisms.

## 2. Materials and Methods

**Figure 1:**
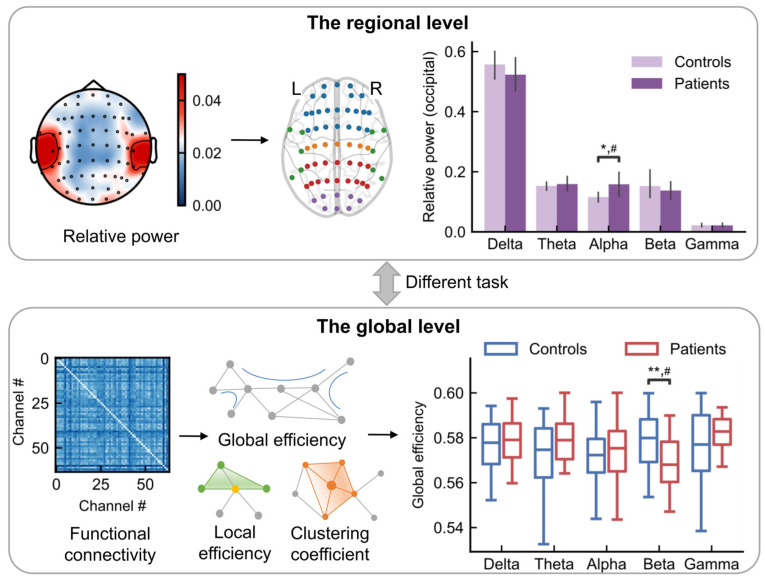
Schematic diagram of EEG data analysis. Following data acquisition and preprocessing, features were extracted in the time-frequency (the regional level) and spatial-frequency (the global level) domains for subsequent statistical comparison between FC patients and healthy controls. **Top:** The analysis in the regional level. Top left: Relative power scalp topography (schematic). Top middle: the EEG electrode positions. Blue, orange, red, purple, and green represents the locations from frontal, central, parietal, occipital and temporal lobes, respectively. Top right: the relative power (schematic) for a given task and brain region for controls and patients. **Bottom:** The analysis in the global level. Bottom left: Functional connectivity matrices. Bottom middle: The network properties, such as global efficiency, local efficiency and clustering coefficient. Bottom right: The statistical comparison between controls and patients. ** *p* < 0.01; * *p* < 0.05; # indicates a medium effect (absolute value of Cohen’s d > 0.5).

This study investigated the EEG signals of FC patients and healthy controls from both time-frequency and spatial domains (Figure 1). For the time-frequency analysis, we computed the relative power of each brain region across five frequency bands (delta, theta, alpha, beta, and gamma) to quantify the spectral activity of distinct brain regions during different tasks. For the spatial analysis, we extracted whole-brain topological features of functional networks to assess the overall information transmission efficiency of the brain. These features were then compared between FC patients and healthy controls to identify differential patterns characterizing FC. Details regarding the subjects, experimental paradigm, data preprocessing, and specific analytical procedures are provided in the following section.

### 2.1. Subjects

The subjects consisted of 21 patients who met Rome IV diagnostic criteria for FC and 37 healthy controls. The FC group included 4 males and 17 females (age range 23–80 years). Patients meeting the following inclusion criteria were enrolled: (1) Except for constipation, the patients had no other physical illnesses, and those with mental disorders such as cognitive impairment were excluded. (2) Prior to the experiment, the FC group was required to abstain for 24 h from consuming psychoactive substances. (3) Did not participate in other clinical trials in the past 3 months.

The HC group included 22 males and 15 females, with an age range of 20–60 years. Healthy controls meeting the following inclusion criteria were enrolled: (1) They were not diagnosed with FC or any other physical and mental conditions. (2) None took any drugs acting on the central nervous system. (3) Had no history of gastrointestinal surgery or head trauma with loss of consciousness.

All participants were right-handed to exclude the impact of this factor on the results. All subjects were recruited from Sir Run Run Shaw Hospital (Hangzhou, China) and were asked to sign the informed consent form.

### 2.2. Experimental Paradigm

**Figure 2:**
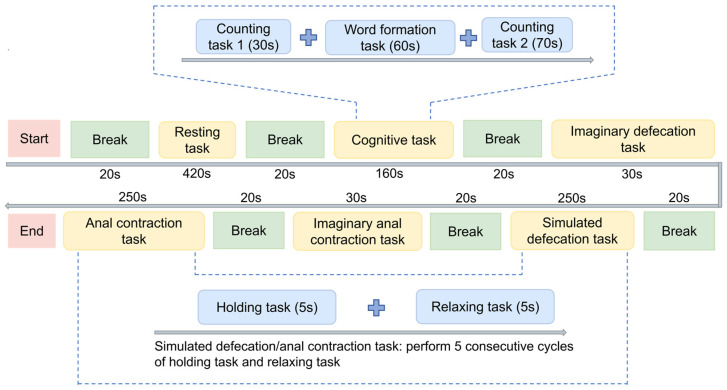
Experimental procedure. The protocol consists of multi-tasks, including resting task, cognitive task (including counting task 1, word formation task, counting task 2), imaginary defecation/anal contraction tasks, simulated defecation task and anal contraction tasks, separated by fixed breaks. Simulated tasks involve 5 cycles of 5 s holding task and 10 s relaxing task.

The study employed a multi-task EEG paradigm (Figure 2) designed to probe brain activity across three functional states: resting, cognitive engagement, and defecation-related sensorimotor control. All tasks were guided by on-screen visual cues, and participants were instructed to remain comfortably seated to minimize movement-related artifacts.

The experimental paradigm incorporated a sequence of tasks systematically organized into distinct procedural segments (Figure 2).

(1) Resting-state baseline: Participants began with a 420 s eyes-open resting-state recording to establish a baseline of intrinsic brain activity.
(2) Cognitive tasks: This 160 s block includes counting task 1 (an arithmetic progression task, counting upward by 7 from 100; 20 trials), a word-formation task (generating bi-character words from given morphemes; 15 trials), and counting task 2 (a prime-number identification task, identifying prime numbers between 1 and 100; 25 trials). These cognitive tasks were designed to assess the participants’ general cognitive engagement.
(3) Defecation-related tasks: Participants performed an imaginary defecation task (30 s; imagining the act of defecation without any actual movement) and a simulated defecation task (250 s; performing a Valsalva maneuver that requires breath-holding and increased abdominal pressure to mimic defecation [21]), which consisted of five consecutive cycles of holding and relaxing tasks. Then, the paradigm included an imaginary anal contraction task (30 s; imagining anal contraction without any actual movement) and an anal contraction task (250 s; performing voluntary anal contraction with kinematic verification via surface electromyography (sEMG)), which also consisted of five cycles of voluntary holding and relaxing tasks. These defecation-related tasks were included to elicit neural activity associated with defecation-related motor control while differentiating between ideomotor representation (imagery) and actual physiological strain. All tasks were performed with eyes open.

### 2.3. EEG Setup and Acquisition of Data

**Figure 3:**
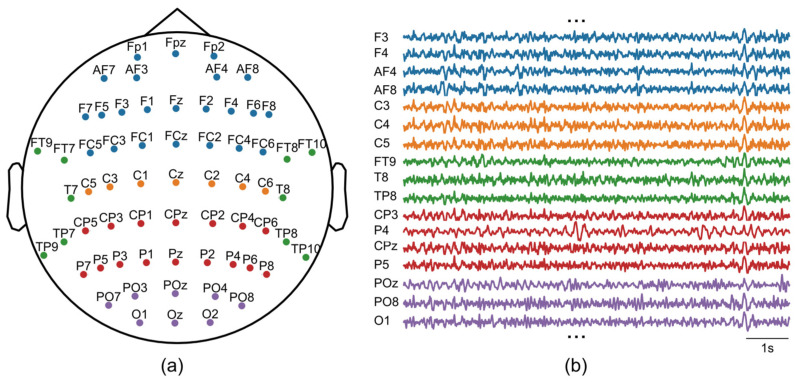
EEG recordings. (**a**) The montage of 64-channel EEG. (**b**) Examples of raw EEG signals.

A 64-channel EEG cap set (GNC-JS05-1064DZ; Greentek, Wuhan, China) was used in conjunction with a supporting 64-channel EEG acquisition system (ML-BCI-64D1; Zhejiang Mailian Medical Technology Co., Ltd., Hangzhou, China) to achieve amplification of EEG signals and synchronous recording. During acquisition, the sampling rate was set to 250 Hz, with the reference electrode placed on the left earlobe and the bias electrode on the right earlobe. Electrode placement adhered to the 10–20 international system montage. A schematic diagram of the real-time EEG signal and 64-channel distribution is shown in Figure 3.

We performed bandpass filtering on raw signals within the range of 0.5–45 Hz, a frequency range that encompasses major EEG bands (delta, theta, alpha, beta, and gamma) while effectively attenuating low-frequency drifts and high-frequency noise. We defined a bad channel as one with prolonged flatlining, where >60% of data points had near-zero amplitude (<1 × 10^−10^ μV). A bad segment was defined by transient flatlining in any channel, detected by the same amplitude threshold. The percentage of detected bad channels was 6.47% ± 9.47% (calculated as the number of bad channels divided by the total number of channels). The percentage of detected bad segments was 1.15% ± 1.27% (calculated as the total duration of bad segments across all channels divided by the product of the number of channels and the total recording duration). The spherical spline interpolation was adopted to reconstruct the bad channel’s data based on the signals from the surrounding good channels, which was implemented using MNE python package [22]. We utilized independent component analysis (ICA) to decompose multichannel EEG signals into statistically independent components (ICs). Based on the ICA results, we employed the ICLabel algorithm [23] to identify and reject artifactual components associated with electrooculographic (EOG), electromyographic (EMG), and electrocardiographic (ECG) activities. Finally, manual inspection was performed to exclude residual artifacts or bad segments that persisted after automated preprocessing. The percentage of removed artifacts was 3.36% ± 6.45% (calculated as the duration of removed artifacts divided by the total recording duration).

In this study, we analyzed the first two minutes of the resting-state recording to ensure signal stability, while for all other task blocks, the entire recorded duration was used for analysis.

### 2.4. Relative Power

To investigate the differences in brain activation levels between patients with FC and healthy controls under various tasks, we calculated the relative power in five different frequency bands (delta: 0.5–4 Hz, theta: 4–8 Hz, alpha: 8–13 Hz, beta: 13–30 Hz, gamma: 30–45 Hz). The relative power of each frequency band was calculated as the ratio of its integrated absolute power to the total integrated power across the 0.5–45 Hz spectrum [24], expressed mathematically as:

(1) $${RP}_{b}=\frac{\sum_{f\in \Omega_{b}}P(f)}{\sum_{k=0.5}^{45}P(f)}$$

where ${RP}_{b}$ denotes the relative power of target band *b* (*b* = δ, θ, α, β, or γ), $\Omega_{b}$ represents the frequency range of band *b*, and P(f) is the power corresponding to frequency f.

By normalizing band-specific power against total spectral power, relative power quantifies the contribution of individual frequency bands to overall neural activity, which helps to control for inter-individual disparities in signal attenuation and volume conduction effects [24].

### 2.5. Topological Features of Brain Networks

To assess potential brain network information transmission deficits in patients with FC, we computed functional connectivity and its topological features. We computed the imaginary part of coherence for all channel pairs across all tasks and frequency bands to derive functional connectivity matrices, a method chosen for its robustness to volume conduction [25]. These matrices were used to construct brain networks, with channels as nodes and connectivity values as edges. To minimize confounding effects of weak connections and avoid the bias of a single threshold, we applied a range of sparsity thresholds (10% to 30% in 5% intervals), retaining the strongest connections for each network [26,27]. For the constructed brain network at each sparsity threshold, the global efficiency, average local efficiency, and average clustering coefficient were extracted as the topological features [12]. These metrics reflect the brain’s information exchange capability during tasks, by quantifying the communication efficiency between brain regions. To obtain a single, integrated, and threshold-independent measure of each topological feature, we calculated the area under the curve (AUC) for each metric across the entire sparsity range [28]. The AUC value, which represents the integral of each topological metric’s trajectory over all thresholds, was subsequently used as the quantitative measure of the corresponding topological feature for all subsequent analyses.

### 2.6. Statistical Analysis

Statistical comparisons between patients with FC and healthy controls were conducted under a standardized hypothesis testing framework. Group differences in EEG features were evaluated using analysis of covariance (ANCOVA), with group entered as the fixed factor and age included as a covariate. To account for multiple comparisons, *p*-values obtained from the ANCOVA were adjusted using the Benjamini–Hochberg false discovery rate (FDR) procedure. FDR correction was applied separately for each task and each frequency band across all brain regions. The significance level was set at α = 0.05. Effect sizes were quantified using Cohen’s d, calculated as the difference between group means divided by the pooled standard deviation. The medium effect was defined as an absolute Cohen’s d greater than 0.5.

## 3. Results

### 3.1. Task-Dependent Alterations of Oscillatory Activity in FC

**Figure 4:**
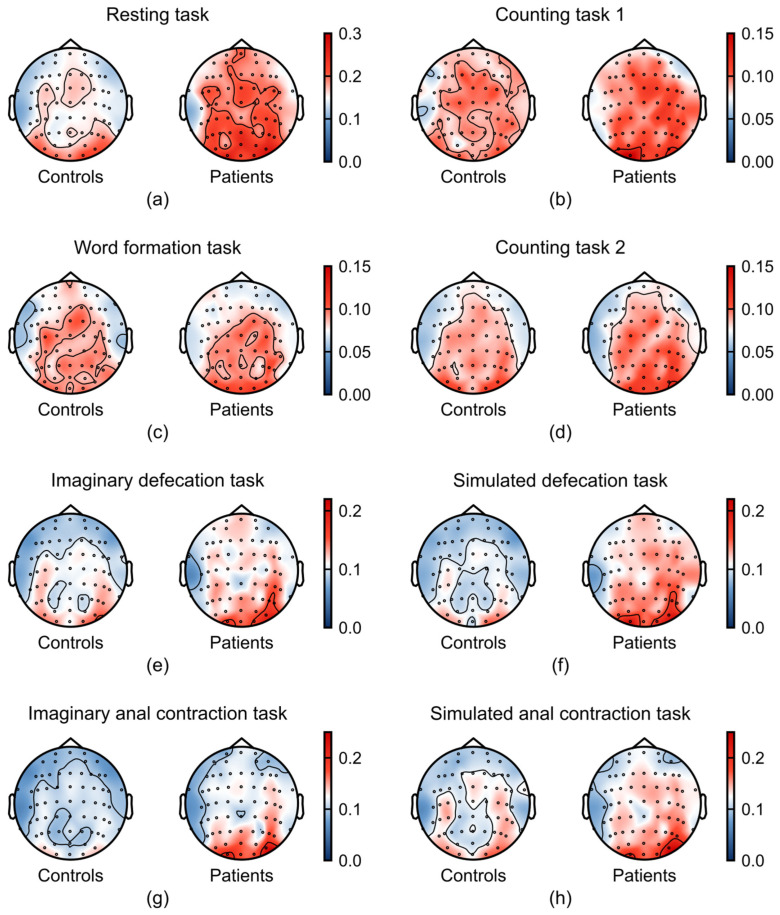
Task-dependent differences in alpha band (8–13 Hz) between patients with FC (n = 21) and healthy controls (n = 37). (**a**–**h**) Average relative power in alpha band across the experimental tasks: (**a**) resting task, (**b**) counting task 1, (**c**) word formation task, (**d**) counting task 2, (**e**) imaginary defecation task, (**f**) simulated defecation task, (**g**) imaginary anal contraction task, and (**h**) anal contraction task.

**Figure 5:**
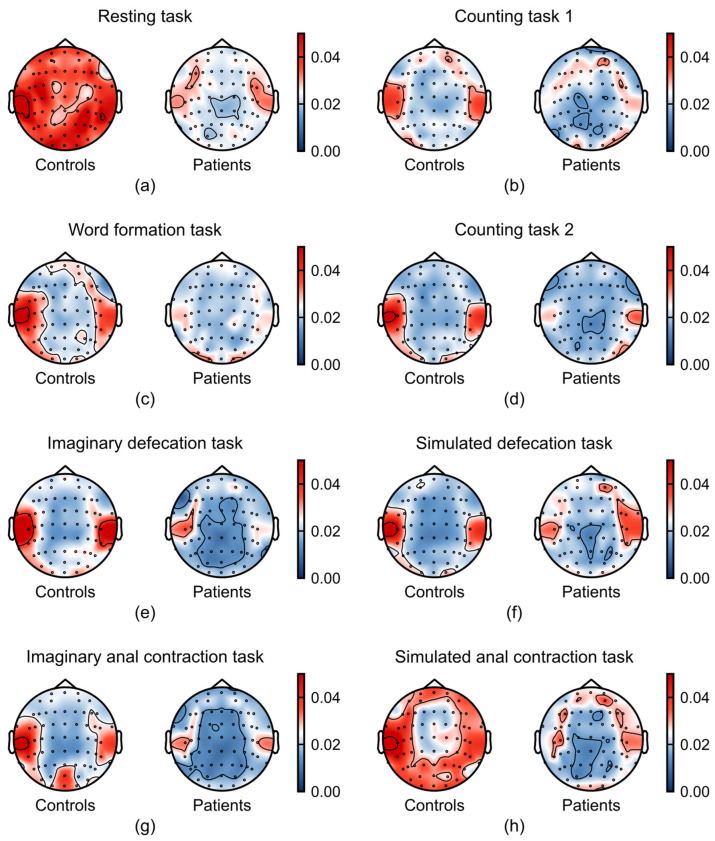
Task-dependent differences in gamma band (30–45 Hz) between patients with FC (n = 21) and healthy controls (n = 37). (**a**–**h**) Average relative power in gamma band across the experimental tasks: (**a**) resting task, (**b**) counting task 1, (**c**) word formation task, (**d**) counting task 2, (**e**) imaginary defecation task, (**f**) simulated defecation task, (**g**) imaginary anal contraction task, and (**h**) anal contraction task.

To visualize the spatial distribution of relative power, we generated topographic maps of relative power for each frequency band and task, comparing FC patients and healthy controls. The topographical maps revealed a divergence in the average relative power between patients with FC and healthy controls, particularly within the alpha (Figure 4) and gamma bands (Figure 5).

As shown in Figure 4, FC patients exhibited a marked increase in alpha-band activity across multiple tasks, particularly during the defecation-related tasks. This enhancement was localized to the parieto-occipital regions, a pattern previously associated with reduced sense of agency (SoA) in prior studies [29,30]. Additionally, increased frontal alpha power during the simulated defecation task suggests reduced frontal cortical engagement in patients.

Gamma power was consistently attenuated in FC patients compared to healthy controls, a difference that was most evident during the resting and anal contraction task (Figure 5). Notably, we found that the differences in EEG signals between the two groups were more prominent in the temporal lobe, a brain region critical for integrating emotional signals and regulating affective responses [31,32]. These findings converge to suggest that diminished gamma oscillations may underlie emotional processing deficits in FC.

### 3.2. Region-Specific Alterations of Oscillatory Activity in FC

To further quantify the differences in oscillatory activity between FC patients and healthy controls, we analyzed the relative power of five frequency bands across different brain regions.

**Figure 6:**
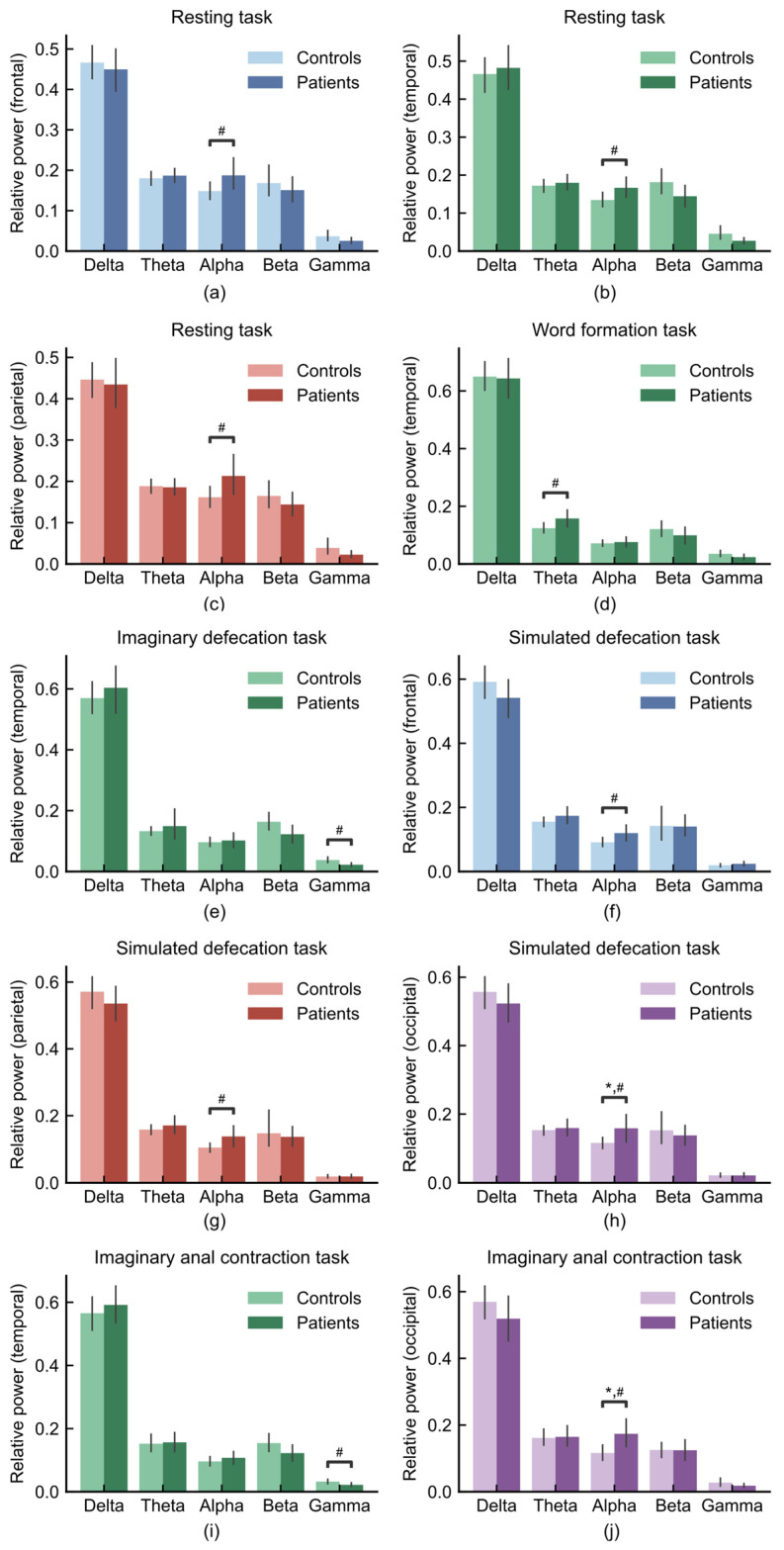
Regional and task-specific oscillatory difference between FC patients (n = 21) and healthy controls (n = 37). (**a**) Frontal lobe during resting task, (**b**) temporal lobe during resting task, (**c**) parietal lobe during resting task, (**d**) temporal lobe during word formation task, (**e**) temporal lobe during imaginary defecation task, (**f**) frontal lobe during simulated defecation task, (**g**) parietal lobe during simulated defecation task, (**h**) occipital lobe during simulated defecation task, (**i**) temporal lobe during imaginary anal contraction task, (**j**) occipital lobe during imaginary anal contraction task. * *p* < 0.05; # indicates a medium effect (absolute value of Cohen’s d > 0.5).

During the resting task, FC patients exhibited higher alpha power in the frontal (Figure 6a; Cohen’s d = 0.53), temporal (Figure 6b; Cohen’s d = 0.54) and parietal lobe (Figure 6c; Cohen’s d = 0.58) compared to healthy controls. In the word formation task, a between-group difference was observed in the theta band, with FC patients showing higher theta power than healthy controls in the temporal lobe (Figure 6d; Cohen’s d = 0.55). During the imaginary defecation task, temporal gamma was lower in patients (Figure 6e; Cohen’s d = −0.66). In the simulated defecation task, patients exhibited increased alpha power in the frontal lobe relative to controls (Figure 6f; Cohen’s d = 0.58). This pattern of alpha enhancement was also observed in the parietal (Figure 6g; Cohen’s d = 0.62) and occipital (Figure 6h; ANCOVA, age-controlled, FDR-corrected *p* = 0.028, Cohen’s d = 0.60) lobe during simulated defecation task. Furthermore, during the imaginary anal contraction task, FC patients exhibited lower temporal gamma (Figure 6i; Cohen’s d = −0.53) and higher occipital alpha (Figure 6j; ANCOVA, age-controlled, FDR-corrected *p* = 0.033, Cohen’s d = 0.71).

### 3.3. The Topological Features of Brain Network in FC

**Figure 7:**
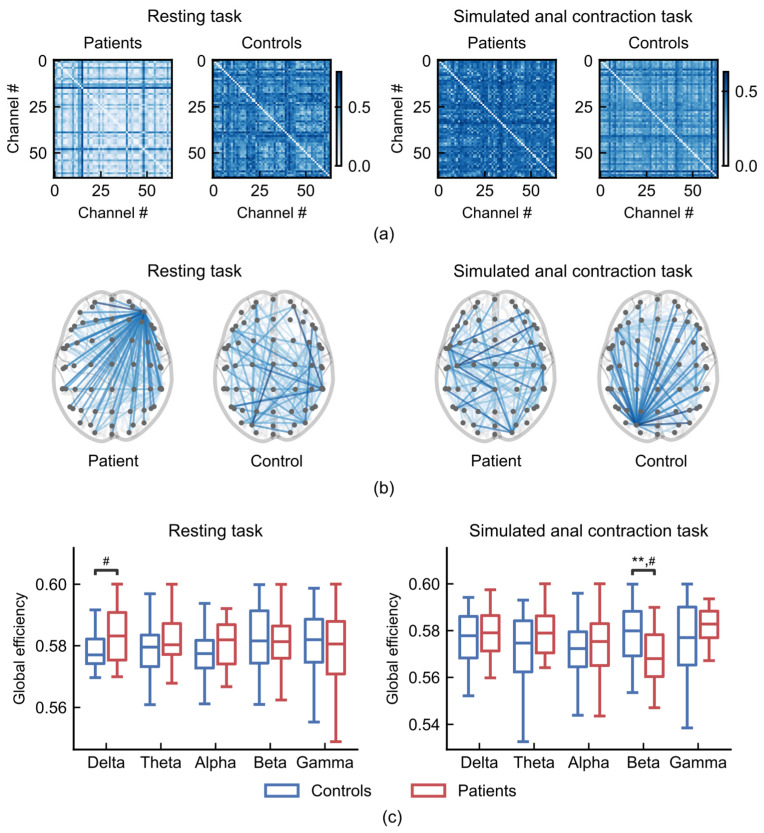
Global efficiency of brain networks during resting (**left**) and anal contraction task (**right**). (**a**) Functional connectivity matrices from a patient (subject 3) and a control (subject 10). (**b**) Representative sparsified functional connectivity networks from a patient (subject 3) and a control (subject 10) (sparsity threshold= 0.1). (**c**) Comparisons of global efficiency between patients with FC (n = 21) and healthy controls (n = 37). ** *p* < 0.01; # indicates a medium effect (absolute value of Cohen’s d > 0.5).

To investigate the difference in global level, we compared the topological features of brain network between FC patients and healthy controls. The topological features, such as global efficiency, was computed from the sparsified functional connectivity networks (Figure 7a,b; see Section 2).

During the resting task, FC patients exhibited stronger hub nodes that connected to more regions than those in healthy controls (Figure 7b, left), resulting in higher global efficiency in the delta frequency band compared to healthy controls (Figure 7c, left; Cohen’s d = 0.60). During the anal contraction task, FC patients exhibited weaker hub nodes (Figure 7b, right), resulting in a significantly lower global efficiency in the beta frequency band compared to healthy controls (Figure 7c, right; ANCOVA, age-controlled, *p* = 0.0054, Cohen’s d = −0.78).

The global efficiency, which quantifies the inverse of the average shortest path length in the network [33], measures the ability of information transmission between brain regions [34]. In patients with FC, the increased global efficiency in the delta band during the resting task may suggest heightened interregional information transfer activity in the resting brain. Conversely, in the simulated anal constriction task, the decrease in global efficiency of patients with FC indicates a reduction in their brain’s ability to control defecation-related movements [35]. Our results demonstrate a dynamic, condition-specific reorganization of functional network topology in FC.

## 4. Discussion

This study investigated the differences in brain activity between FC patients and healthy controls across resting, cognitive, and defecation-related tasks. We observed that FC patients elevated alpha oscillations during resting and defecation-related tasks, diminished temporal gamma during defecation-related tasks, and increased temporal theta during the word formation task. Furthermore, brain network topology analysis identified significant alterations in delta-band networks during the resting task and beta-band networks during the anal contraction task.

The brain–gut axis provides a bidirectional communication pathway between the gastrointestinal enteric nervous system and the central nervous system, and plays a critical role in both the development and recovery of FC [6,36]. Evidence suggests that increased gut activity is associated with enhanced bodily self-consciousness, which encompasses the SoA over one’s bodily functions [37]. Given the established inverse correlation between SoA and parieto-occipital alpha-band activity [29], our finding of elevated alpha power in these regions during resting and defecation-related tasks may reflect a reduced sense of agency during defecatory control, which could contribute to impaired gastrointestinal motility. In addition, FC patients also showed increased alpha relative power in the frontal lobe during the simulated defecation task. Since alpha activity is generally considered an inverse index of cortical engagement [38], this increase suggests reduced frontal cortical involvement during the sensory-motor integration required for defecation. This pattern is consistent with previous reports of abnormal dorsolateral prefrontal and anterior cingulate activation in FC patients during rectal distension [39], further supporting the presence of disrupted top-down regulation in defecatory control.

The observed gamma suppression in patients with FC may also originate from the brain–gut axis dysregulation. Specifically, patients with FC exhibit intestinal flora disruption [40], which may impair N-methyl-D-aspartate subtypes of glutamate receptors (NMDARs) function through metabolites, leading to decreased synchrony in Parvalbumin positive interneurons (PV+) neurons and disrupting the coordination of gamma oscillations [41]. Simultaneously, gamma oscillations are involved in the central processing of intestinal signals and the motor control related to bowel movements [42]. Consequently, a self-reinforcing cycle may form between reduced gamma oscillations and constipation, further exacerbating constipation symptoms.

The temporal lobe serves as the core brain region underlying affective processing [31,32,43]. Our results revealed reduced gamma-band power and increased theta-band power in the temporal lobe of FC patients across resting, cognitive, and defecation-related tasks. This pattern of oscillatory suggests specific impairments in emotional processing and cognitive regulation, which is consistent with prior reports of cortical morphometric abnormalities in brain regions involved in emotional processing in constipated individuals [35], as well as deficits in sensory integration and emotional processing [44].

Delta oscillations are typically associated with unconscious processing, sensory integration, and default-mode network (DMN) activity [45,46]. The increased global efficiency in the delta band observed during the resting state may reflect enhanced connectivity between DMN core regions (e.g., the dorsal medial prefrontal cortex (DMPFC) and precuneus (PCUN)) and the inferior frontal gyrus (IFG)—a key region for emotional regulation [47]. This hyper-connectivity could reinforce the over processing of gut-related sensory signals and negative emotions (e.g., anxiety or depression), which are common in patients with FC [48,49]. Beta oscillations play a critical role in motor planning, execution, and sensorimotor integration [50,51]. The reduced global efficiency observed during the anal contraction task indicates that the brain’s capacity to synchronize motor commands with visceral sensory feedback is impaired. This finding aligns with both the insufficient local connectivity in motor-execution-related regions (e.g., the paracentral lobule) [13] and the prevalent symptom of pelvic floor muscle dyssynergia in patients with FC [52,53].

According to Ukhtomsky’s dominant theory, a highly excited focus can merge within the central nervous system at a given moment and possesses the capacity to suppress other unrelated reflex activities [54,55]. For FC patients, their altered brain activity can be understood as follows: pathological physiological activity related to gut function establishes a persistent pathological dominant focus within the central nervous system. This entrenched abnormal focus of excitation chronically interferes with and suppresses normal cognitive processing, emotional regulation, and sensorimotor control functions.

In summary, this study identifies a distinct profile of task-dependent oscillatory in FC patients, encompassing alpha, temporal gamma, temporal theta, and delta/beta network efficiency. These convergent findings strongly support a model of widespread brain–gut axis dysfunction, contributing to the neural underpinnings of FC’s core symptoms. This electrophysiological profile may inform future biomarker development and targeted interventions. However, this work has several limitations. First, the relatively small sample size (21 FC patients and 37 healthy controls), may limit the generalizability of our findings. Future studies with larger and more diverse cohorts are needed to validate our conclusions. Second, the uneven age distribution in our dataset prevented a systematically investigation of the potential impact of age on FC symptoms and brain activity patterns. Age is known to influence both gastrointestinal motility [56] and brain network dynamics [57,58], and its role in modulating the relationship between FC pathophysiology and neural activity remains unclear.

Future studies should extend our findings by employing age-stratified sampling and incorporating measures of constipation severity, task-based behavioral metrics, and standardized psychological assessments to further validate the observed neural signatures. Beyond unimodal EEG, integrating complementary modalities such as magnetoencephalography (MEG) or functional near-infrared spectroscopy (fNIRS) could enrich the spatial and hemodynamic characterization of brain–gut interactions. Moreover, the identified electrophysiological alterations suggest specific mechanistic targets for neuromodulation—for instance, transcranial electrical stimulation could be explored to modulate cortical excitability and counteract alpha-gamma dysregulation, though such approaches require systematic validation in dedicated intervention trials.

## 5. Conclusions

In conclusion, this study explores brain activity and network topological properties in patients with FC by comparing them with healthy controls across resting, cognitive, and defecation-related tasks. Using EEG relative power analysis and graph theory-based network topology assessments, we identified distinct neural activity in FC patients. The key findings of our study are that FC patients exhibited elevated alpha power in frontal and parieto-occipital regions during the resting and defecation-related tasks, decreased gamma-band power in the temporal lobe during the resting and defecation-related tasks, and increased theta-band power in the temporal lobe during the word formation task, as well as altered global efficiency in delta-band networks during rest and in beta-band networks during anal contraction.

Our results provide direct neurophysiological evidence that functional brain dysregulation represents a central manifestation of brain–gut axis dysfunction in FC. Specifically, during resting, elevated alpha power and reduced gamma signify reduced cortical engagement, potentially hindering its readiness to response. The increased temporal theta power during the word formation task indicates elevated cognitive load in FC patients, suggesting that their brains require greater neural effort than healthy controls to achieve comparable performance, resulting in reduced neural efficiency. During defecation-related tasks, which requires precise sensorimotor and emotional integration, the observed pattern of elevated alpha and reduced gamma oscillations indicates reduced cortical engagement and a diminished sense of agency. This oscillatory profile signifies a core breakdown in the brain–gut dialogue necessary for top-down defecatory control. As for the global-level network findings, the enhanced delta-band efficiency suggests hyper-connectivity among default-mode and emotion-regulation regions, consistent with the excessive processing of gut-related sensations and negative emotions frequently reported in FC. In contrast, reduced beta-band efficiency during anal contraction reflects impaired sensorimotor integration and weakened coordination of motor-execution regions, aligning with pelvic-floor dyssynergia. These results reveal disruptions in emotional processing, cognitive regulation, and motor control-related information transmission in FC patients, suggesting that central nervous system dysfunction plays a integral role in FC pathophysiology.

This work establishes a foundation for understanding FC from a perspective of brain activity, incorporating both specific brain region functions and whole-brain network dynamics. The identified electrophysiological patterns potentially serve as biomarkers to inform future research on diagnostic and therapeutic strategies for FC.

## Data Availability

The raw data of this study are available from the corresponding author upon reasonable request. The analysis code is publicly available on GitHub at: https://github.com/yinawei-lab/Constipation-EEG-analysis, accessed on 23 December 2025.
